# Ribosome nascent chain complexes of the chloroplast-encoded cytochrome b_6_ thylakoid membrane protein interact with cpSRP54 but not with cpSecY

**DOI:** 10.1007/s10863-014-9598-0

**Published:** 2015-01-06

**Authors:** Małgorzata Piskozub, Bożena Króliczewska, Jarosław Króliczewski

**Affiliations:** 1 Faculty of Biotechnology, University of Wrocław, Fryderyka Joliot-Curie 14a, 50-383 Wroclaw, Poland; 2 Department of Animal Physiology and Biostructure, Wrocław University of Environmental and Life Sciences, Wrocław, Poland

**Keywords:** Cytochrome b_6_, Membrane protein, Thylakoid membrane, RNC, cpSecY, cpSRP54

## Abstract

We analysed the interplay between the cpSecY, cpSRP54 and the chloroplast-encoded cytochrome b_6_ via isolation of chloroplast ribosome nascent chain complexes and the use of cross-linking factors, antibodies and mass spectroscopy analyses. We showed that the cytochrome b_6_ nascent polypeptide complex is tightly associated with ribosomes and that the translation of cytochrome b_6_ was discontinuous. The causes of ribosome pausing and the functional significance of this phenomenon may be related to proper protein folding, insertion into thylakoid membranes and the association of cofactors during this process. It was also found that cpSecY was not in the vicinity of cytochrome b_6_ intermediates during the elongation process and does not act with mature cytochrome b_6_ after translation. Using the approach of cross-linking during elongation of the cytochrome b_6_ protein, we showed that cpSRP54 interacts strongly with the elongating nascent chain.

**Electronic supplementary material:**

The online version of this article (doi:10.1007/s10863-014-9598-0) contains supplementary material, which is available to authorized users.

## Introduction

In the chloroplast the major complexes responsible for electron transport, i.e., photosystem I, photosystem II, the cytochrome b_6_f complex and the ATP synthase complex contain subunits that are encoded from both the nuclear and chloroplast genomes. During the formation of these functional complexes, numerous regulatory factors are required for the coordinated transport and assembly of the subunits. To date import into or across the thylakoid membrane of nuclear encoded proteins has been thought to occur through four independent precursor-specific thylakoid transport pathways (cpTat, cpSec, cpSRP and the spontaneous integration pathway) (Schuenemann 2007). As chloroplasts are of prokaryotic origin, it is not surprising that several of the chloroplast targeting and translocon components share not only a strong homology with those in E. coli but also pronounced differences from bacterial transport mechanisms.

In bacteria, most inner membrane proteins are targeted by a signal recognition particle (SRP)-dependent mechanism, whereas in chloroplasts the SRP-dependent posttranslational transport of nuclear encoded thylakoid membrane proteins seems to be limited to members of the light-harvesting chlorophyll binding proteins (LHCPs) (Schünemann 2004). The cpSRP pathway requires a heterodimer of cpSRP43 and cpSRP54 subunits in the stroma and then interacts with cpFtsY. LHCP is inserted into the thylakoid membrane by the integral membrane protein Alb3, a member of the YidC⁄Oxa1 family (Moore et al. 2003). In the stroma, cpSRP54 is present in two distinct pools. One pool is associated with cpSRP43 and is active in the posttranslational targeting of LHCP, whereas the other pool is associated with 70S ribosomes suggesting a role for cpSRP54, but not cpSRP43, in the sorting of chloroplast-encoded proteins (Schuenemann et al. 1998). Alb3 has been implicated in the folding and assembly of the D1 protein into photosystem II. The D1 protein is a chloroplast-encoded protein with five transmembrane segments. It is synthesized by chloroplast ribosomes and interacts with cpSRP54 during synthesis (Nilsson et al. 1999). The YidC/Alb3 protein can either function in concert with the Sec translocase or can function independently of the Sec translocase, where it is possibly involved in the insertion, folding or assembly of chloroplast-encoded thylakoid membrane proteins (Moore et al. 2003; Yi and Dalbey 2005; Yuan et al. 2010; Wang and Dalbey 2011). The D1 protein is inserted into the thylakoid membrane by cpSecY, as demonstrated via cross-linking and co-immunoprecipitation experiments which showed that nascent chains of D1 interact with the cpSecY, and subsequently assemble into PSII (Zhang et al. 2001). It has also been shown that cpSRP54 interacts with the first transmembrane domain of the D1 protein, as long as the nascent chain is still attached to the ribosome, thus indicating the role of cpSRP54 in co-translational protein transport (Nilsson and van Wijk 2002).

In contrast, chloroplast-encoded cytochrome *f*, with cleavable lumenal transit peptide, was shown to be cotranslationally inserted into the membrane requiring cpSecA, the Sec translocon and ATP (Röhl and van Wijk 2001).

The cpSec pathway, which evolved from the general secretory pathway involved in the export of Sec-dependent proteins to the periplasm in bacteria and in *E. coli* consisting minimally of SecA, SecE and SecY (Akimaru et al. 1991) is also involved in the co-translational insertion of SRP-dependent proteins into the plasma membrane (Houben et al. 2002; Yuan et al. 2010). In chloroplasts, our knowledge of the cpSec pathway is limited, with current models being mainly based on homology to the bacterial Sec system and little is known about the role of the chloroplast Sec translocase in the insertion of proteins into the thylakoid membrane (Aldridge et al. 2009).

One of the thylakoid proteins, cytochrome b_6_ is a multispanning membrane core subunit of the cytochrome b_6_f complex which binds one heme molecule covalently and two heme molecules non-covalently (Kurisu et al. 2003; Stroebel et al. 2003). This protein, encoded by the chloroplast gene *petB*, like many other thylakoid integral proteins, operates with uncleaved and unknown targeting signals for thylakoid integration (Zak et al. 1997; Zhang et al. 2001). In recent reports we have shown that apocytochrome b_6_ from spinach can be heterologously expressed as a fusion protein in *E. coli* and the expressed fusion protein integrates into the *E. coli* inner membrane and a protein assembles with spectroscopic characteristics typical for cytochrome b_6_ (Kroliczewski and Szczepaniak 2002; Kroliczewski et al. 2005). The fusion of polytopic cytochrome b_6_ to maltose binding protein (MBP) directs the cytochrome to the Sec-dependent pathway and topogenic signals in the amino acid sequence of the nascent chain of the chloroplast cytochrome b_6_ protein are recognized by the *E. coli* Sec translocon, leading to the integration of this protein into the bacterial inner membrane; however with an opposite orientation compared to that in the thylakoid membrane (Kroliczewski et al. 2005). *E. coli* CM124 cells with depleted SecE (a subunit of SecYE translocon) show that apocytochrome b_6_ expressed in cells is found only in the cytoplasm with no signal deriving from apocytochrome b_6_ being detected in the membrane fraction and the insertion of polytopic cytochrome b_6_ into the cytoplasmic bacterial membrane is totally dependent on the presence of an artificially added N-terminal presequence (Kroliczewski et al. 2005, 2011).

In vitro assays for the post-translational spontaneous insertion of the chloroplast-encoded cytochrome b_6_ by isolated pea thylakoids have also been studied. Both native or denatured cytochrome b_6_ isolated from *Synechocystis sp*. PCC 6803, overexpressed in *E. coli* and synthetic cytochrome b_6_ with the signal sequence from OE33 were used. For all these case we have been unable to demonstrate import of cytochrome b_6_ into isolated thylakoids membrane either with or without stromal extract (Kroliczewski and Piskozub 2011). In other reports, Dreher et al. have shown that transmembrane cytochrome b_6_ spontaneously assembles in *E. coli* as well as in a biological membrane (Dreher et al. 2007). Such unspecific interaction of cytochrome with a bacterial membrane was also observed in a previous study, but in that instance during expression apocytochrome b_6_ was degraded (Kroliczewski et al. 2005).

However, to obtain a more complete picture of protein transport to the thylakoid membrane further experimental studies are required to elucidate the exact mechanistic details of the chloroplast Sec and spontaneous pathways and to unravel the question of the role of Alb3 in protein insertion.

Since current results do not explain the insertion of cytochrome b_6_ into the thylakoid membrane in this study we decided to analyze the interplay between cpSecY and the chloroplast-encoded cytochrome b_6_ protein by isolation of ribosome nascent chain (RNC) complexes from chloroplasts and by the use of crosslinking factors and antibodies for immunoprecipitation together with mass spectroscopy (MS), electrophoresis and Western blot analyses.

## Materials and methods

### Plant materials

Seeds of pea (*Pisum sativum*, cv Calvedon) were germinated on moistened filter paper at 25 °C in darkness for 4 days. Seedlings were then grown hydroponically within a 10 h photoperiod at a photon flux density of 300 μmol·m^−2^·s^−1^ for 7–10 days in a growth chamber at 23 °C. For all the experiments, fully developed leaves were harvested 2 h after the lights were turned on.

### Isolation of intact chloroplasts

Approximately 50 g of pea leaves were ground in 250 ml of grinding media (HB) containing 330 mM sorbitol, 5 mM ascorbic acid, 0.05 % BSA, 2 mM EDTA, 1 mM MgCl_2_, 50 mM HEPES, pH 7.6. The homogenate was passed through four layers of muslin and for the purification of plastids a “crude plastid fraction” was sedimented by centrifugation 1000×*g*, 5 min. The sediments were gently resuspended in a small amount of ice-cold buffer A (330 mM sorbitol, 2 mM DL-Dithiothreitol (DTT), 50 mM HEPES, pH 8.0) and layered on top of the Percoll™ (Pharmacia)-buffer A gradients. Intact chloroplasts were isolated by Percoll™ gradient centrifugation according to the method by Morgenthaler et al. with some modifications (10,300 rpm, 10 min, 4 °C in 40 ml tubes of the Beckman rotor SW 32 Ti) (Morgenthaler et al. 1975). The isopycnically banded plastids were collected, washed twice (2000×*g* 2 min, 4 °C) and resuspended in buffer A at a chlorophyll concentration of ~1 mg/ml. Total chlorophyll content was measured according to Arnon (Arnon 1949).

### Isolation of ribosome-nascent chain complexes

RNCs were isolated from intact chloroplast using the method by Zhang et al. with some modifications (Zhang et al. 2001). Intact pea chloroplasts were lysed and thylakoids solubilized with 2.5 % (*w/v*) dodecyl-D-maltoside (DM) in the lysis buffer 50 mM Hepes-KOH, pH 7.5, 5 mM magnesium acetate (MgOAc), 50 mM potassium acetate (KOAc), 2 mM DTT and a mixture of protease inhibitors (2 μg/ml antipain, and 2 μg/ml leupeptin) at 0.5 mg of chloroplasts/1 ml for 30 min on ice. The elongation inhibitor chloramphenicol was added to a final concentration of 250 μg/ml to stabilize the ribosomes further. RNCs were collected by centrifugation in 4-ml tubes through a 1.0 M sucrose cushion in ice-cold lysis buffer at 51,300 rpm, 1 h of the Beckman rotor SW 60 Ti. To increase the purity of RNC complexes with cytochrome b_6_ and increase the detection signal, additional purification of RNC complexes by sedimentation through sucrose cushion gradients were performed. To follow cytochrome b_6_ elongation and detections of translation intermediates, RNasin® Ribonuclease inhibitors were used and chloramphenicol was omitted.

### Cross-linking

Chloroplast RNCs were cross-linked on ice under optimized conditions using the heterobifunctional cleavable cross-linker *N*-succinimidyl-3-[2-pyridyldithio]propionate (SPDP, Pierce) or irreversible homo-bifunctional sulfhydryl cross-linker bismaleimidohexane (BMH, Pierce). Freshly prepared chloroplast RNCs were suspended in 50 mM Hepes-KOH, pH 7.2, 5 mM MgOAc, 50 mM KOAc, and centrifuged for 2 min at 5000×*g* to remove any aggregated material. Cross-linking reactions of interacting proteins were then performed on ice for 30 min by adding the SPDP or BMH to a final concentration of 1 mM. The protease inhibitors antipain, leupeptin and pepstatin were also added to a final concentration of 1 μg/ml. After incubation, BMH cross-linkers were quenched by the addition of 150 mM β-mercaptoethanol and kept for at least 45 min on ice. Products cross-linked by SPDP were also cleaved with a reducing agent (20 mM DTT) and subjected to further immunoprecipitation.

### Immunoprecipitation

The method by Zhang et al. with some modifications were used for immunoprecipitations (Zhang et al. 2001). For denatured immunoprecipitation, the samples were denatured in 1 % SDS in 50 mM Tris–HCl (pH 8.0) in the presence of protease inhibitor cocktail (cOmplete ultra-Tablets, Mini, EDTA-free, Roche) and heated for 15 min at 37 °C. Subsequently, samples were diluted five times with 1 % Triton X-100, 150 mM NaCl, 50 mM Tris–HCl, pH 7.5, 2 mM EDTA, and a cocktail of protease inhibitors (Roche).

For native immunoprecipitation, the samples were solubilized in 1 % DM, 150 mM NaCl, 50 mM Tris–HCl, pH 7.5, and a mixture of protease inhibitors. Appropriate antibodies were added and incubated for 16 h at 4 °C with gentle mixing. Subsequently, protein A- Sepharose CL-4B (GE Healthcare) was added and incubation was continued for an additional 4 h at 4 °C. Finally, the beads were washed five times in 1 % Triton X-100, 150 mM NaCl, 2 mM EDTA and 50 mM Tris–HCl, pH 7.5, resuspended in SDS solubilization buffer, and used for electrophoresis analysis.

For double immunoprecipitation, the first immunocomplexes were centrifuged at 3000 rpm and the supernatant was removed. The pellet was resuspended in SDS lysis buffer (20 mM Tris pH 7.5, 50 mM NaCl, 1 % SDS) and boiled for 5 min. Quick spin at room temperature to pellet beads was performed. Than the supernatant was used for the next immunoprecipitation with a second antibody cross-linked to protein A-Sepharose CL-4B beads. The samples were incubated at room temperature for 4 h. The Sepharose beads were then washed four times with 50 mM Tris–HCl, pH 7.5, 1 % Triton X-100, 150 mM NaCl, 1 mM EDTA and once with 50 mM Tris–HCl (pH 7.5). Precipitated antibody-antigen complexes were eluted with SDS solubilization buffer containing β-mercaptoethanol and separated by electrophoresis followed by immunoblotting (Bonifacino et al. 2001).

### Mass spectroscopy

The MS analyses were performed by the Mass Spectroscopy Service at IBB PAN Warsaw, Poland. Proteins co-purifying with cpSecY or cytochrome b_6_ were analyzed in electrophoresis, stained with Coomassie solution and subjected to Western blotting. Furthermore, protein found in a stained band, was excised from a polyacrylamide gel and analyzed by MS. Prior to analysis the gel slices containing the desired spots, were subjected to a standard procedure during which proteins were reduced with 100 mM DTT for 30 min at 56 °C, alkylated with iodoacetamide in darkness for 45 min at room temperature and digested overnight with trypsin. The resulting peptides were eluted from the gel with 0.1 % formic acid (FA) and 2 % acetonitrile (ACN). The resulting peptide mixtures were applied to RP-18 pre-column (Waters, Milford, MA) using water containing 0.1 % FA as a mobile phase and then transferred to an nano-HPLC RP-18 column (Waters, Milford MA) using ACN gradient (0–30 % ACN in 45 min) in the presence of 0.1 % FA at a flow rate of 250 nl/min. The column outlet (Nano-Acquity Waters LC system) was coupled directly to the ion source of an LTQ FTICR mass spectrometer (Thermo Electron Corp., San Jose, CA) working in the regime of data-dependent MS to tandem mass spectrometry (MS/MS) switch. A blank run ensuring absence of cross-contamination from previous samples preceded each analysis.

After preprocessing of the raw data with Mascot Distiller software (version 2.1.1, Matrix Science), output lists of precursor and product ions were compared to the National Center for Biotechnology database using Mascot database search engine (version 2.1, Matrix Science) (Perkins et al. 1999). Proteins hit with a combined peptide score higher than 59 were considered significant.

### Protein analysis

Protein concentration was determined using the bicinchoninic acid (BCA) reagent according to the manufacturer’s instructions (Sigma). SDS-PAGE electrophoresis in the presence of SDS was performed in 12 % polyacrylamide gels according to Laemmli (Laemmli 1970). Denaturation of samples was performed by mixing the protein sample with solubilization buffer (300 mM Tris–HCl, pH 6.8, 60 % glycerol, 12 mM EDTA, 12 % SDS, 864 mM β-mercaptoethanol, 0.05 % bromophenol blue) in a v\v ratio of 5:1. For membrane proteins, in a solubilization buffer, 2-mercaptoethanol was changed for 480 mM DTT. Protein bands were detected by staining with Coomassie Brilliant Blue.

Tricine/Tris SDS–PAGE. A small pore 16.5%T separation gel was overlayered by a 10%T spacer gel (approximately 0.5 cm) and a 4 % T stacking gel T indicates the total percentage concentration of acrylamide and bisacrylamide (Schagger and von Jagow 1987).

BN-PAGE was performed essentially as described in (Schägger and von Jagow 1991) with the following modifications. RNC complexes were isolated and then solubilized with 1.6 % dodecyl maltoside in 1.5 M amino caproic acid and 75 mM BisTris (pH 7.0). Electrophoresis was run on 4–16 % polyacrylamide gradient gel on a Bio-Rad Protean II Mini gel system. Gels were run at 35 V for 30 min and at 300–450 V (10 mA) for 3 h. When the tracking dye reached two-third of the gel height, the electrophoresis was interrupted and the blue cathode buffer (50 mM Tricine, 15 mM BisTris, pH 7.0, 0.02 % CBB G-250) was replaced by colorless cathode buffer (50 mM Tricine, 15 mM BisTris, pH 7.0). Electrophoresis was stopped when the tracking line of CBB G-250 dye had left the edge of the gel. After BN gel electrophoresis, the protein complexes were visible as sharp Coomassie blue stained bands. One dimensional BN strips could be further analyzed by Western blot analysis on two-dimensional 10 % SDS-PAGE gels after denaturating incubation in 1 % SDS/1 % β-mercaptoethanol solution.

### Western blot

Western blot analysis was performed by the standard techniques, using detection horseradish peroxidase-conjugated goat anti-rabbit and a semidry blotting system. The PCR-amplified cpSRP54 cDNA fragment was inserted into pGS-21a (GeneScript, USA) and the resulting plasmid was used for the expression of cpSRP54 in *Escherichia coli* BL21 (DE3) cells. The overexpressed protein was purified and used for the generation of antibodies in rabbits. Antibodies raised against 21 C-terminal residues (CRAEIISQKYKNIELYDFDKY) of pea cpSecY and against N-terminal residues of cytochrome b_6_ (SKVYDWFEERLEIQ and IQAIADDITSKYVPPHVN) were prepared in rabbits injected with the synthetic peptide coupled to ovalbumin (GeneScript, USA).

For control experiments, antibodies against the N-terminal residues (58–86) of the D1 protein were used. The used antibody raised against cpSecY recognized a 35 kDa protein in the stromal thylakoid membranes, which are the sites of protein translocation and integration (Hashimoto et al. 1997). Membrane was also stripped and reprobed with another antibody as described using Thermo Scientific Restore Western Blot Stripping Buffer (Thermo Scientific) (Kaufmann and Kellner 1998). Protein bands were detected by incubating with horseradish peroxidase-conjugated antibodies and visualized with chemiluminescence reagent (Thermo Scientific, SuperSignal West Femto Substrate).

## Results

In this study, evaluation of the interaction of cytochrome b_6_ with cpSecY and cpSRP54 proteins was set out via immunological and MS analysis of RNC complexes.

**Fig. 1:**
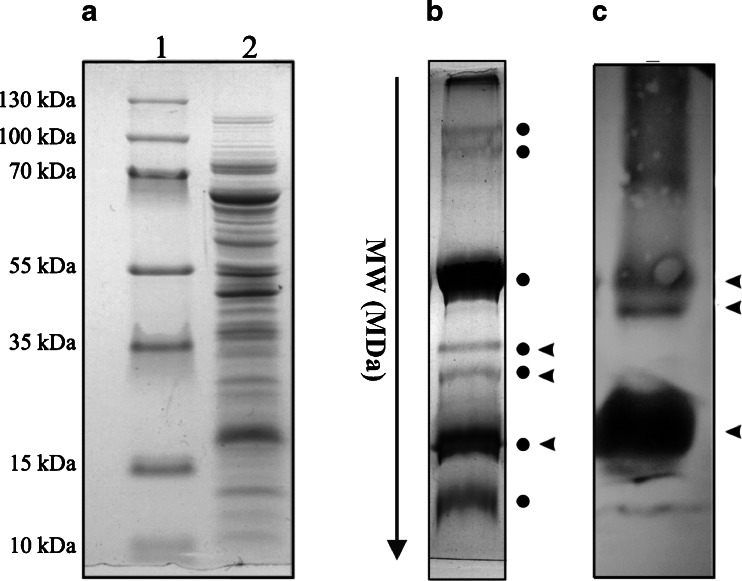
Cross-linked RNC complexes isolated from intact chloroplasts. Purified ribosome-nascent chain complexes bearing cytochrome b_6_ were cross-linked with interacting components using BMH. **a** SDS-PAGE of cross-linked RNC complexes. Lane 1, molecular mass standards; lane 2, RNC preparation from pea chloroplast. **b** 1-D BN-PAGE gel of cross-linked RNC complexes stained by CBB R-250. Gel bands subjected to PMF are indicated by *filled circle.* **c** Western blot analysis of the gel prepared as in (**b**), detection by antibody against NH_2_-terminus of cytochrome b_6_. The *arrowhead* indicates the bands that contained cross-linked cytochrome b_6_

Before experiments were started, tests to check the specificity and reactivity of all antibodies were conducted. The results of antibody specificity against cytochrome b_6_, cpSecY and against the D1 protein (control protein) were presented on Fig. SP1A. For the purpose of the test a total chloroplast protein isolated from pea was used. In addition antibody cross-linked to protein A Sepharose CL-4B was also used to test immunoprecipitation of interesting proteins (Fig. SP1B). Antibody raised against the 18 C-terminal amino acids of spinach cpSecY recognized one protein of ∼ 35 kDa in the thylakoid membrane (Fig. SP1B, lane 2). Western blot (Fig. 1SP) and mass spectroscopic (data not shown) analyses of immunoprecipitated protein have shown that cytochrome b_6_, D1 protein and cpSecY from total chloroplast protein were efficiently precipitated. In addition, any weak cross-reaction of antibodies with other proteins isolated from the chloroplast was observed (Fig. SP1A).

### Cytochrome b_6_ elongation intermediates

Isolated chloroplasts generally do not synthesize adequate amounts of proteins to enable a study of their mode of integration and the underlying conditions. Biochemical and structural studies of co-translational folding, targeting and translocation depend on an efficient methodology to prepare RNCs. In particular, structural studies of interactions between various factors and macromolecular complexes with ribosomes carrying a nascent polypeptide chain, are hampered by technical difficulties entailed in obtaining large amounts of pure ribosomes and RNC complexes. The second problem is ensuring an adequate level of protein detection and choosing the detection method.

To follow cytochrome b_6_ translation elongation and the concomitant translocation and insertion into the thylakoid membrane, we isolated RNC complexes (Fig. 1a, lane 2, SDS-PAGE of RNC complexes) and next cross-linked via BMH, and then quantitatively immunoprecipitated the cytochrome b_6_ nascent chains with an excess of antibody against N-terminal residues of cytochrome b_6_.

The cytochrome b_6_ RNC complexes were detected in isolated and cross-linked RNCs (Fig. 1). In addition, the composition of separated complexes can be determined directly via MS. When subjecting the gel band from the RNC complexes separated by 1-D BN-PAGE (see Fig. 1b, black circle) to MS, prevalently ribosome proteins were also identified (Table 1). The MS data were further verified by immunoblotting of the 1-D BN-PAGE gel with anti-cytochrome b_6_. The black arrowhead indicates the gel bands that contained cross-linked cytochrome b_6_ (Fig. 1b and c).

**Table 1:** Identification of proteins located in isolated RNC-cytochrome b_6_ complexes by mass spectroscopy and peptide mass fingerprinting

Sequence record processed by NCBI	Protein name
gi|42559164	50S ribosomal protein L3
gi|1350625	50S ribosomal protein L1
gi|400986	50S ribosomal protein L15
gi|27446517	50S ribosomal protein L2
gi|118489599	Unknown, similar to rplE region of 50S ribosomal protein L5
gi|132819	50S ribosomal protein L24
gi|5305933	cytochrome b_6_ apoprotein [Pisum sativum]
gi|3914841	30S ribosomal protein S4,
gi|62287174	30S ribosomal protein S7,
gi|62287174	30S ribosomal protein S7,
gi|133829	30S ribosomal protein S17
gi|133913	30S ribosomal protein S2,

Table presented selected examples of ribosomal protein located in the isolated RNCs. We identified more than twenty ribosomal protein

**Fig. 2:**
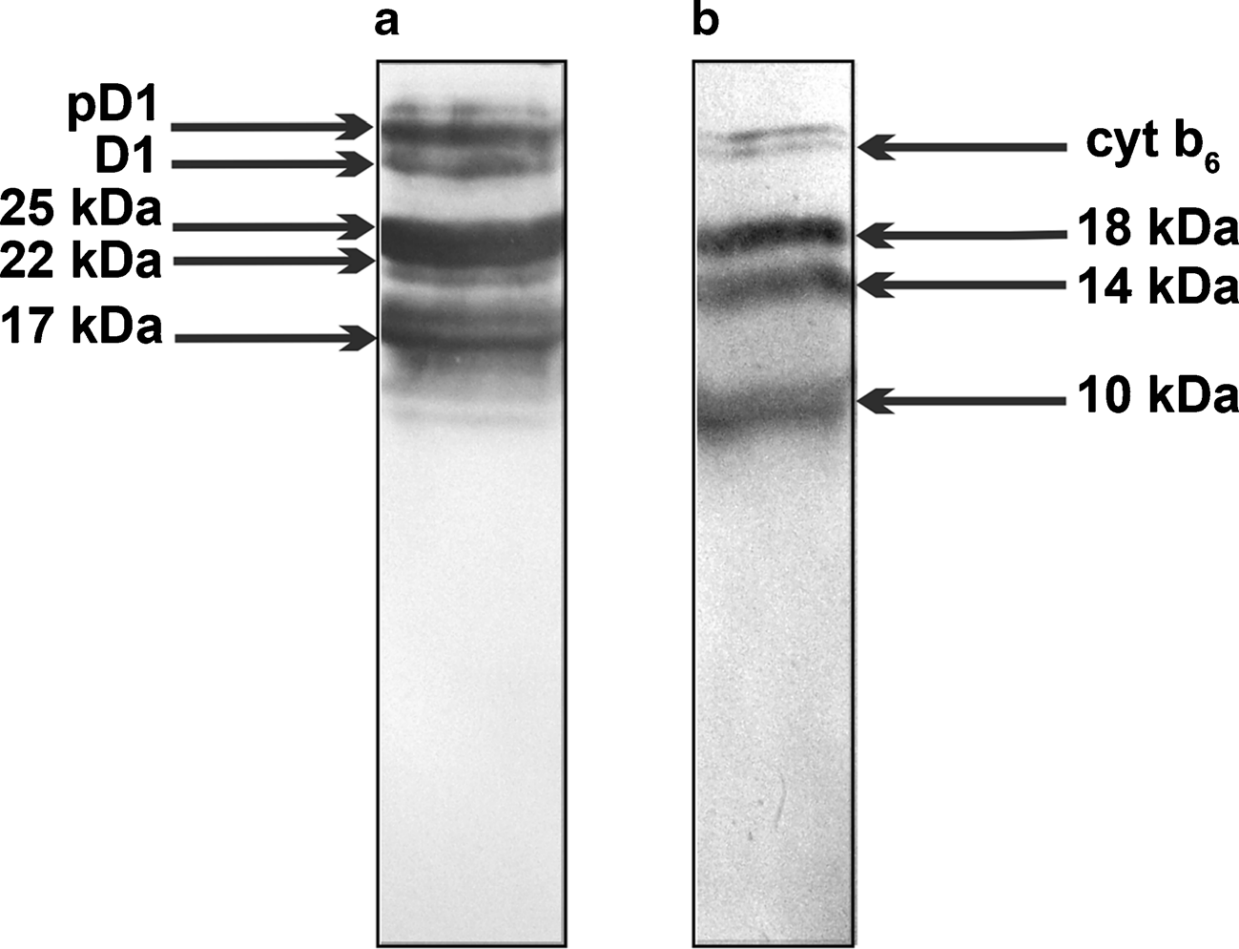
Western blot analysis of (**a**) D1 protein translation elongation in intact chloroplasts. The thylakoid membrane-bound RNCs were solubilized with SDS, and D1 elongation intermediates were immunoprecipitated with an excess of specific N-terminal D1 antibody. **b** Western blot of several cytochrome b_6_ protein translation elongation intermediates in intact chloroplasts. The thylakoid membrane-bound RNCs were solubilized with SDS, and cytochrome b_6_ elongation intermediates were immunoprecipitated with an excess of N-terminal b_6_ antibody. Precipitated RNCs were separated by SDS-PAGE and visualized by Western blot. The D1 elongation intermediates are indicated as well as the mature (D1) and precursor (pD1) forms. The cytochrome b_6_ elongation intermediates of 18, 14, and 10 kDa as well as the mature (cytb_6_) forms are indicated. The molecular mass (kDa) of D1 and cytochrome b_6_ intermediates and their identification was carried out using mass spectrometry (Table SP1)

The control protein in the experiment was the D1 protein. The D1 protein has the highest turnover rate of the known thylakoid membrane proteins and in vivo it is constantly synthesized and inserted into the mature thylakoid membrane system (Zhang et al. 2001). Data from in vivo and cell-free experiments suggested that synthesis of proteins may proceed discontinuously due to translational pausing. During elongation of the D1 protein, ribosomes pause at several distinct sites, generating pausing intermediates 17–25 kDa (Zhang et al. 1999, 2000). The D1 intermediates were precipitated with an excess of D1 antibody (Zhang et al. 2001). We found (Fig. 2a) that several of the Dl translation intermediates co-sediment with polysomes, and this results was in accordance with previous investigations (Kim et al. 1991; Zhang et al. 1999).

Because regulation of translation elongation can be expected to lead to the accumulation of translation intermediates on membrane-bound ribosomes, the authors searched for such translation intermediates (pausing products) of cytochrome b_6_ by immunoblotting the polysomal fractions with an N-terminal cytochrome b_6_ antibody.

To characterize the RNC complexes containing cytochrome b_6_ the thylakoid membrane-bound RNCs were solubilized with SDS and cytochrome b_6_ elongation intermediates were immunoprecipitated with an excess of N-terminal b_6_ antibody and then were separated by SDS-PAGE and visualized via Western blot (Fig. 2b). The proteins extracted from the gel were analyzed using mass spectroscopy and identified. Results of identification of cytochrome b_6_ intermediates proteins in Western blot bands by mass spectroscopy and peptide mass fingerprinting (PMF) are depicted in Table SP1. The cytochrome b_6_ elongation intermediates of 18, 14, and 10 kDa we identified using Western blot and MS (Fig. 2b and Table SP1). Anti-cytochrome b_6_ antibodies also recognized a very weak 6-kDa protein signal which was detectable using Western blot with great difficulty. This band was also analyzed with MS and additional cytochrome b_6_ translation intermediate was found (Table SP1).

### Interaction of the cytochrome b_6_ nascent chain with thylakoid proteins

Chloroplast-encoded thylakoid membrane proteins were synthesized on thylakoid membrane-bound ribosomes. Recent data indicate an important role for SecY in bacterial and eukaryotic endoplasmic reticulum membrane protein biogenesis (Mothes et al. 1997; Zhang et al. 2001). In addition, the cpSecY complex is involved in the posttranslational translocation of nuclear-encoded membrane proteins (Mori et al. 1999) and also was found to be associated with ribosomes (Zhang et al. 2001). So it was intriguing to test the involvement of cpSecY in cytochrome b_6_ chloroplast biogenesis.

**Fig. 3:**
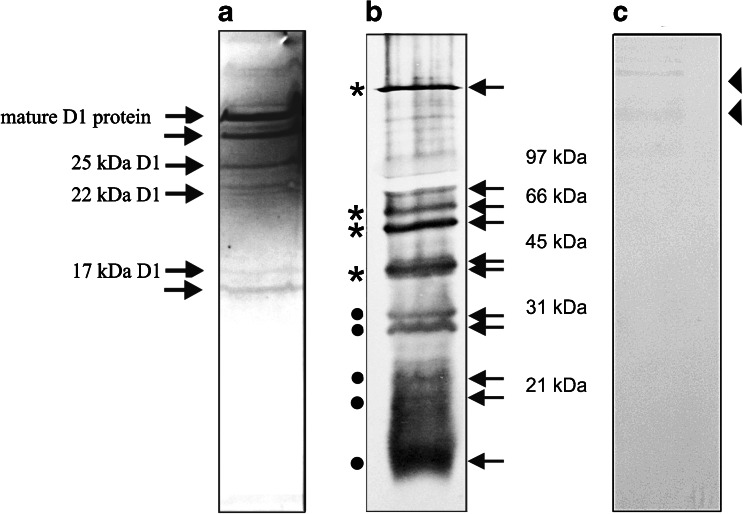
Western blot analysis of RNC–D1 complexes and RNC–cytochrome b_6_ complexes. **a** D1 nascent chain was isolated and a homo-bifunctional cross-linker (BMH) was added. The samples were immunoprecipitated with antibody against D1 protein, solubilized with SDS, and subjected to further immunoprecipitation with anti-cpSecY. The D1 elongation intermediates as well as the mature D1 are indicated by *arrows*. **b** Western blot analysis of the cross-linking (BMH) of isolated RNCs followed by immunoprecipitation with antiserum directed against the N-terminal part of cytochrome b_6_. Gel bands subjected to PMF are indicated by arrows. The bands marked with filled stars contain cytochrome b_6_ intermediates. **c** Western blot analysis of the cross-linking of isolated RNC-cytochrome b_6_ complexes immunoprecipitated with antibody directed against the N-terminal part of cytochrome b_6_, solubilized with SDS and subjected to further immunoprecipitation with anti-cpSecY. A very weak signal marked by triangle was observed. MS analysis of corresponding gel bands from SDS-PAGE was performed

To identify chloroplast proteins interacting with the cytochrome b_6_ RNCs, a cross-linking approach, followed by immunoprecipitation with specific antibodies (an antibody against cytochrome b_6_, D1, or cpSecY) was applied. Freshly prepared RNCs were purified from intact pea chloroplast and then incubated with BMH. Cross-linked products with BMH were immunoprecipitated with appropriate antiserum (against D1 protein or against cytochrome b_6_) after denaturing with SDS. The precipitated products were subjected to further immunoprecipitation with anti-cpSecY. After the second immunoprecipitation, precipitated products were separated on Tricine PAGE and analyzed with Western blot (Fig. 3). Initially, examination of the interaction of soluble stromal proteins with the D1 nascent chain emerging out of the ribosome tunnel was performed. Full-length D1 protein and intermediates synthesized from *psbA* transcripts in the pea chloroplast were observed and developed stable and D1 was co-immunoprecipitated with cpSecY (Fig. 3a).

Figure 3 also shows Western blot analysis of the cross-linking of isolated RNCs with antiserum directed against the N-terminal part of cytochrome b_6_ (Fig. 3b) followed by second immunoprecipitatation with cpSecY antibody (Fig. 3c). Before the second immunoprecipitation, a specific interaction identified via Western blot was observed between ribosome proteins and the nascent chain of cytochrome b_6_ (Fig. 3b). No significant immunoprecipitation of the above-mentioned nascent chain complexes was found after the second precipitation with cpSecY antiserum (Fig. 3c). A very weak signal (Fig. 3c, marked by triangle) was observed in Western blots, which could suggest the possible presence of cpSecY in immunoprecipitated RNC-cytochrome b_6_ complexes. For this reason, the MS analysis of corresponding bands excised from SDS–PAGE gels was performed. However, the analysis did not confirm these observations.

**Fig. 4:**
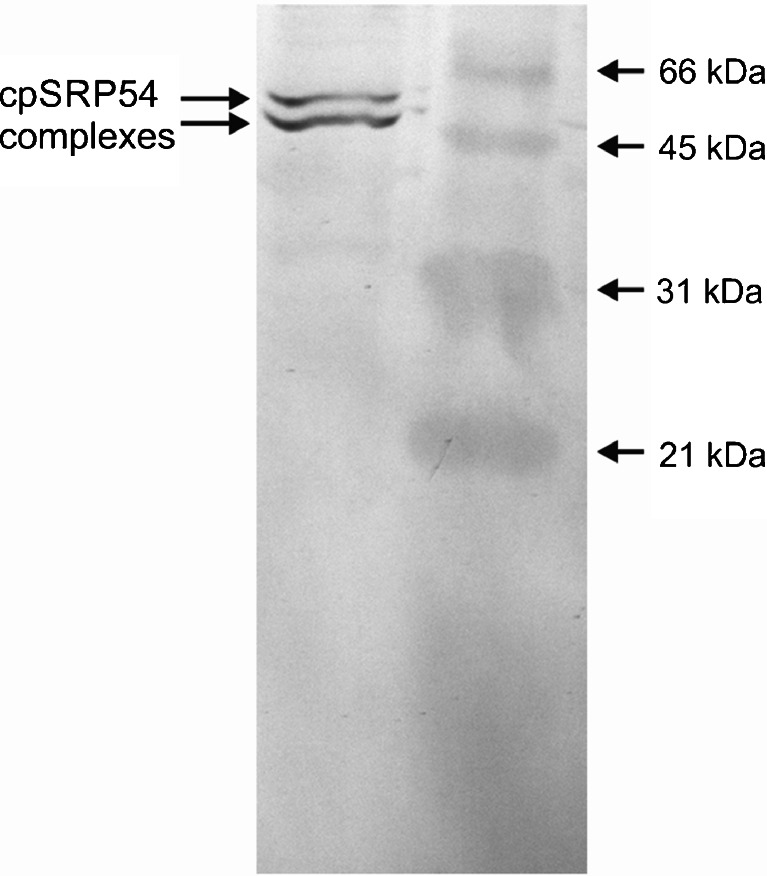
Western blot analysis of the cross-linking of isolated RNC’s followed by immunoprecipitatation with antiserum directed against the N-terminal part of cytochrome b_6._ For immunodetection, polyclonal antibody against cpSRP54 was used. Lane 1, RNCs after cross-linking and immunoprecipitation; lane 2, molecular mass standards

To identify soluble components interacting with the cytochrome b_6_ RNCs (Fig. 3b) with mass spectroscopy with PMF, the protein found in the stained band corresponding to the band in Western blot (Fig. 3b, marked by the arrow) was excised from a polyacrylamide gel and analyzed by MS and then the data were processed using Mascot Distiller software. cpSRP54 was one of the dominant proteins found during the database search. Mascot search results of cpSRP54 cross-linked to 6 kDa cytochrome b_6_ and 10 kDa cytochrome b_6_ are presented in Tables 2 and 3, respectively. The MS data were further verified by immunoblotting. Western blot analysis of the cross-linking of isolated RNCs followed by immunoprecipitatation with antiserum directed against cytochrome b_6_ are presented on Fig. 4. For immunodetection antibody against cpSRP54 was used.

**Table 2:** Mascot Search Results^a^ of cpSRP54 cross-linked to 6 kDa cytochrome b_6_. gi|3746964 Mass: 61448 Score: 911, Queries matched: 41 emPAI: 1.07. Signal recognition particle 54 kDa subunit

Mr(expt)	Mr(calc)	ppm	Miss	Score	Expect	Peptide
701.4048	701.4072	−3.35	0	58	0.0034	R.GGAALSVK.E
951.5363	951.5389	−2.79	0	(56)	0.0017	K.IVHDELVK.L
951.5368	951.5389	−2.22	0	(52)	0.0044	K.IVHDELVK.L
951.5371	951.5389	−1.87	0	(48)	0.011	K.IVHDELVK.L
951.5375	951.5389	−1.45	0	(56)	0.0021	K.IVHDELVK.L
951.5377	951.5389	−1.32	0	72	4.4e-05	K.IVHDELVK.L
951.5378	951.5389	−1.22	0	(66)	0.0002	K.IVHDELVK.L
1015.5897	1015.5913	−1.63	1	(48)	0.019	K.LKGEEVLTK.D
1072.6106	1072.6128	−2.09	1	(50)	0.012	K.LKGEEVLTK.D + Carbamidomethyl (K)
1072.6106	1072.6128	−2.03	1	(51)	0.0093	K.LKGEEVLTK.D + Carbamidomethyl (K)
1072.6107	1072.6128	−2.00	1	(52)	0.0078	K.LKGEEVLTK.D + Carbamidomethyl (K)
1072.6112	1072.6128	−1.51	1	(54)	0.0045	K.LKGEEVLTK.D + Carbamidomethyl (K)
1072.6112	1072.6128	−1.47	1	57	0.0022	K.LKGEEVLTK.D + Carbamidomethyl (K)
1110.6369	1110.6397	−2.54	0	51	0.0043	R.GVKPDQQLVK.I
1260.6439	1260.6462	−1.88	0	72	0.00013	K.FVESASSKPGPR.G
1380.7972	1380.7977	−0.34	0	54	0.002	R.ALLEADVSLPVVR.R
1380.7981	1380.7977	0.33	0	(41)	0.036	R.ALLEADVSLPVVR.R
1406.7505	1406.7479	1.85	0	76	3.2e-05	R.ILGMGDVLSFVEK.A
1424.7171	1424.7259	−6.19	0	(45)	0.039	R.ERPELLAESPER.R
1424.7245	1424.7259	−0.98	0	62	0.00079	R.ERPELLAESPER.R
1424.7297	1424.7259	2.67	0	(54)	0.0048	R.ERPELLAESPER.R
1424.7310	1424.7259	3.61	0	(47)	0.022	R.ERPELLAESPER.R
1426.6026	1426.6075	−3.38	0	(45)	0.011	R.MEDLEPFYPDR.M + Oxidation (M)
1426.6040	1426.6075	−2.44	0	60	0.00033	R.MEDLEPFYPDR.M + Oxidation (M)
1426.6063	1426.6075	−0.80	0	(44)	0.016	R.MEDLEPFYPDR.M + Oxidation (M)
1426.6064	1426.6075	−0.76	0	(56)	0.00095	R.MEDLEPFYPDR.M + Oxidation (M)
1426.6070	1426.6075	−0.29	0	(52)	0.0026	R.MEDLEPFYPDR.M + Oxidation (M)
1426.6073	1426.6075	−0.11	0	(52)	0.0024	R.MEDLEPFYPDR.M +Oxidation (M)
1426.6074	1426.6075	−0.07	0	(46)	0.01	R.MEDLEPFYPDR.M + Oxidation (M)
1426.6074	1426.6075	−0.03	0	(46)	0.0094	R.MEDLEPFYPDR.M + Oxidation (M)
1426.6078	1426.6075	0.21	0	(56)	0.001	R.MEDLEPFYPDR.M + Oxidation (M)
1426.6080	1426.6075	0.41	0	(41)	0.03	R.MEDLEPFYPDR.M + Oxidation (M)
1426.6101	1426.6075	1.88	0	(52)	0.0028	R.MEDLEPFYPDR.M + Oxidation (M)
1508.8914	1508.8926	−0.84	0	(98)	5e-08	K.SGPTVILLAGLQGVGK.T
1508.8936	1508.8926	0.66	0	(45)	0.0087	K.SGPTVILLAGLQGVGK.T
1508.8946	1508.8926	1.32	0	(70)	2.8e-05	K.SGPTVILLAGLQGVGK.T
1508.8955	1508.8926	1.93	0	104	9.6e-09	K.SGPTVILLAGLQGVGK.T
1508.8959	1508.8926	2.17	0	(75)	7.3e-06	K.SGPTVILLAGLQGVGK.T
1508.9016	1508.8926	5.91	0	(45)	0.0069	K.SGPTVILLAGLQGVGK.T
1691.8641	1691.8665	−1.42	0	(105)	4.2e-08	R.FVQSVSDQAVGMGVIR.G
1707.8611	1707.8614	−0.21	0	112	1.3e-08	R.FVQSVSDQAVGMGVIR.G + Oxidation (M)

^a^Ions score is −10*Log(P), where P is the probability that the observed match is a random event. Individual ions scores > 41 indicate identity or extensive homology (*p* < 0.05). Protein scores are derived from ions scores as a non-probabilistic basis for ranking protein hits. The others proteins fund in exanimated samples except cytochrome b_6_ have total score from 1 to 50

**Table 3:** Mascot Search Results^a^ of cpSRP54 cross-linked to 10 kDa cytochrome b_6_. gi|3746964 Mass: 61448 Score: 485, Queries matched: 16 emPAI: 0.55. Signal recognition particle 54 kDa subunit

Mr(expt)	Mr(calc)	ppm	Miss	Score	Expect	Peptide
960.4301	960.4335	−3.53	0	40	0.056	K.DNIAEPMR.D + Oxidation (M)
1074.4801	1074.4829	−2.61	0	59	0.00073	R.QEDAEDLQK.K
1424.7254	1424.7259	−0.36	0	62	0.00079	R.ERPELLAESPER.R
1424.7266	1424.7259	0.51	0	(61)	0.00097	R.ERPELLAESPER.R
1424.7266	1424.7259	0.52	0	(55)	0.0039	R.ERPELLAESPER.R
1424.7299	1424.7259	2.79	0	(58)	0.0019	R.ERPELLAESPER.R
1426.6051	1426.6075	−1.67	0	43	0.021	R.MEDLEPFYPDR.M + Oxidation (M)
1508.8928	1508.8926	0.14	0	(80)	2.4e-06	K.SGPTVILLAGLQGVGK.T
1508.8948	1508.8926	1.45	0	(72)	1.7e-05	K.SGPTVILLAGLQGVGK.T
1508.8960	1508.8926	2.22	0	(73)	1.1e-05	K.SGPTVILLAGLQGVGK.T
1508.8962	1508.8926	2.35	0	(50)	0.0024	K.SGPTVILLAGLQGVGK.T
1508.8964	1508.8926	2.52	0	99	3e-08	K.SGPTVILLAGLQGVGK.T
1508.8993	1508.8926	4.39	0	(63)	0.00011	K.SGPTVILLAGLQGVGK.T
1707.8628	1707.8614	0.82	0	115	6.9e-09	R.FVQSVSDQAVGMGVIR.G + Oxidation (M)
1777.8113	1777.8152	−2.20	1	(44)	0.029	K.ATEVMRQEDAEDLQK.K + Oxidation (M)
1777.8114	1777.8152	−2.17	1	52	0.0054	K.ATEVMRQEDAEDLQK.K + Oxidation (M)

^a^Ions score is −10*Log(P), where P is the probability that the observed match is a random event. Individual ions scores > 41 indicate identity or extensive homology (*p* < 0.05). Protein scores are derived from ions scores as a non-probabilistic basis for ranking protein hits. The others proteins fund in exanimated samples except cytochrome b_6_ have total score from 1 to 50

A large amount of cpSRP54 was found to interact with 6 kDa cytochrome b_6_ intermediates. A significant but smaller amount of cross-linked cpSRP54 was immunoprecipitated with cytochrome 10 kDa, but no precipitation was observed with cytochrome b_6_ 14 and 18 kDa intermediates. Apart from the cross-linked products that contained cytochrome b_6_, a number of other cross-linked bands can be observed (Fig. 3b, marked by black circle). In these bands, unknown proteins were identified by mass spectroscopy.

### Interaction of cpSecY with cytochrome b_6_

**Fig. 5:**
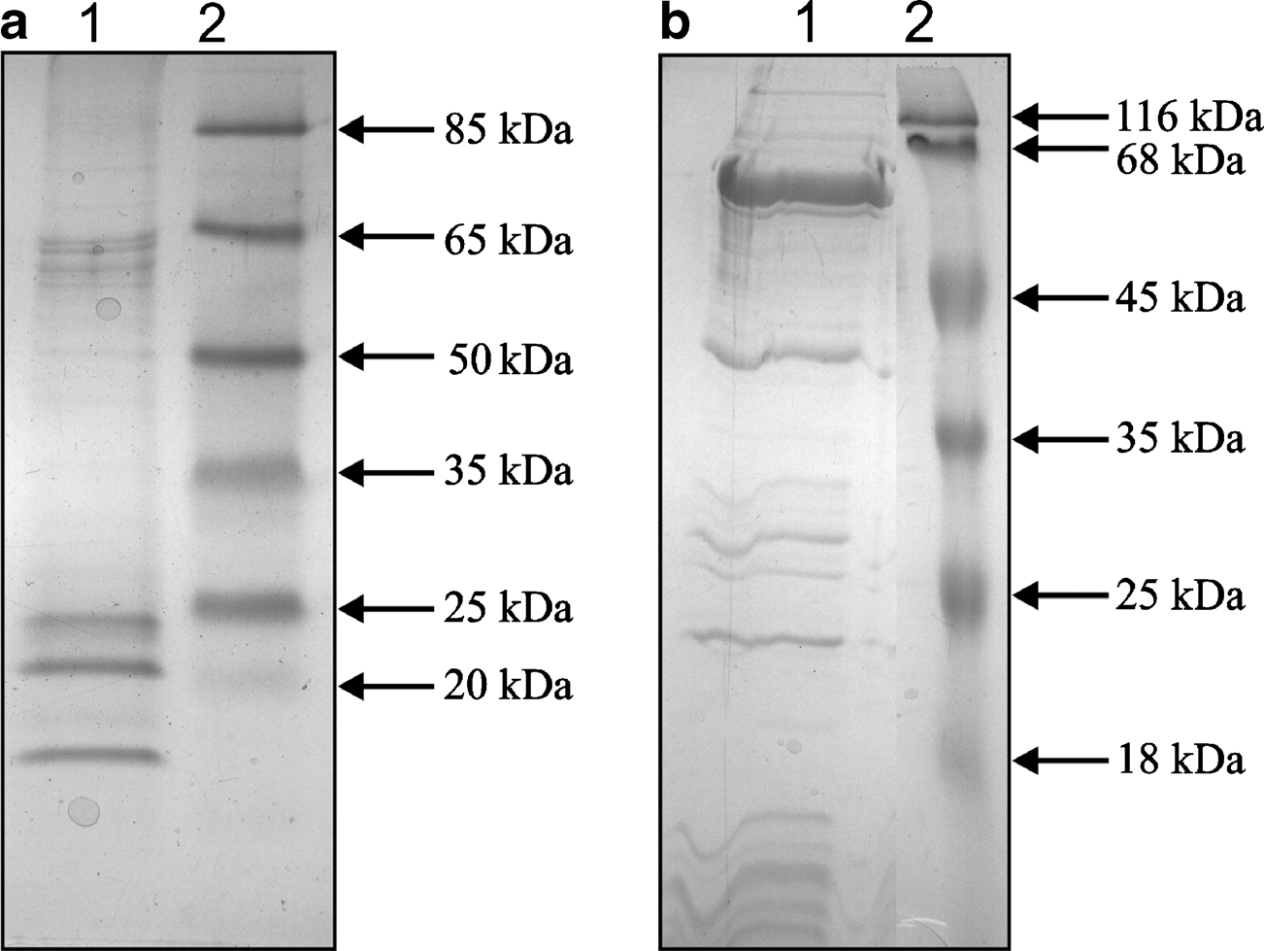
SDS-PAGE analysis of RNC complexes isolated from intact chloroplasts after cross-linking. **a** Lane 1, RNCs after crosslinking with BMH; lane 2, molecular mass standards. **b** Lane 1, RNCs after crosslinking with SPDP; lane 2, molecular mass standards

To elucidate the possible interaction of cpSecY with cytochrome b_6_, isolated RNCs were purified by double sucrose cushion centrifugation from contaminating protein complexes. After purification, RNCs and any associated factors were incubated in buffer with cross-linkers BMH or SPDP. With both cross-linkers, a clear and distinct cross-linking pattern was observed (Fig. 5), whereas without the addition of cross-linkers, no cross-linked bands were observed (not shown). The cross-linking pattern achieved with the two cross-linkers was quite different, indicative of specific cross-linking (Fig. 5).

**Fig. 6:**
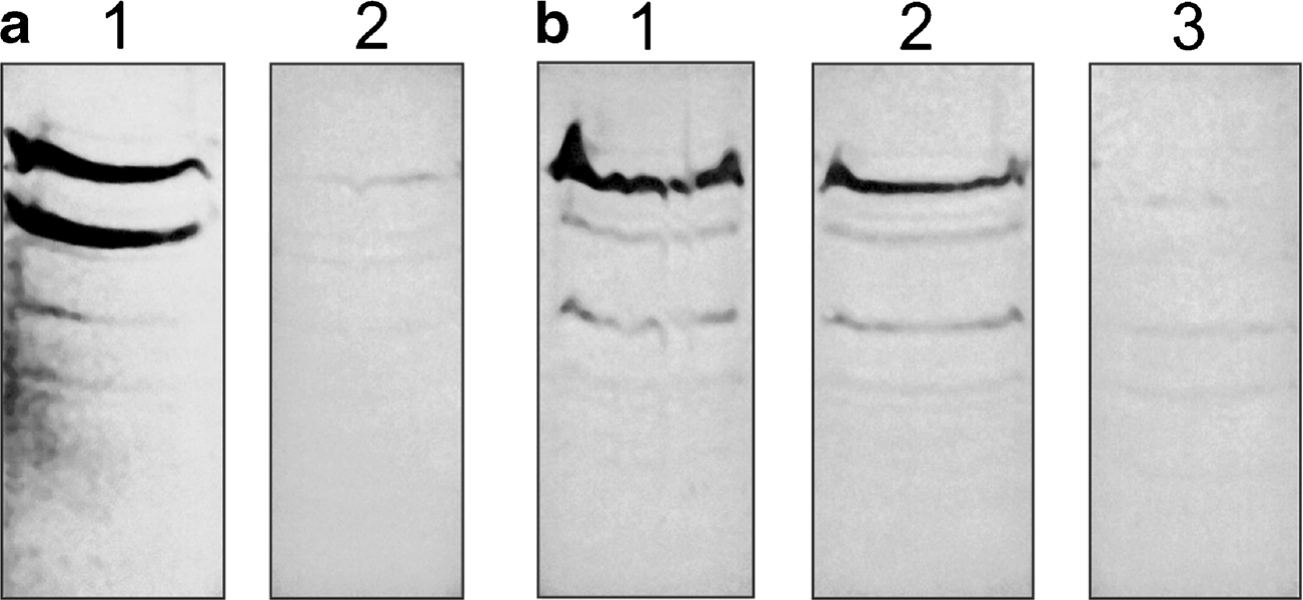
Western blot analysis of cross-linked RNC complexes. **a** Lane 1, RNCs after cross-linking with BMH; lane 2, test for complete removal of the HRP label. The membrane was striped and incubated with new SuperSignal West Femto Substrate Working Solution and exposed to film. No signals were detected using a 5-min exposure. **b** Lane 1, RNCs after cross-linking with BMH; lane 2, RNCs after cross-linking with BMH and next membrane was stripped and reprobed with anti-cytochrome b_6_ antibody; lane 3, RNCs after cross-linking with BMH, and next membrane was stripped and reprobed with anti-cpSecY antibody

To further verify the interaction of cytochrome b_6_ with cpSecY Western blotting, striping and reprobing analysis were performed. Western blot analysis against cytochrome b_6_ showed the anti-cytochrome b_6_ antibodies recognizing the N-terminus of cytochrome b_6_ in isolated RNC-cytochrome b_6_ complexes (Fig. 6a, lane 1). After the first detection the membrane was washed and submerged in stripping buffer at 37 °C for 30 min with occasional agitation. To determine if any primary antibody remained (negative control), the Western blot pieces was stained again. The membrane should be washed several times, blocked, incubated with secondary antibody, and then reincubated with chemiluminescent substrate (Fig. 6a, lane 2). After determining that the membrane is properly stripped (Fig. 6a, lane 2), the second immunoprobing experiment may be performed (Fig. 6b). Western blots of RNC complexes were detected with SuperSignal West Femto Substrate. The first blot used polyclonal rabbit anti-cytochrome b_6_ primary antibody with an HRP-secondary conjugate (Fig. 6b, lane 1). The same blot was stripped for 5 min at room temperature in Restore Western Blot Stripping Buffer and then re-probed with purified anti-cytochrome b_6_ primary antibody (positive control, Fig. 6b, lane 2) or anti-cpSecY primary antibody (Fig. 6b, lane 3) with the HRP-secondary conjugate. As presented in Fig. 6b, the cytochrome b_6_ intermediates in RNCs could be detected with the cytochrome b_6_ antibody, but we did not observe a cpSecY association with cytochrome b_6_ molecules.

**Fig. 7:**
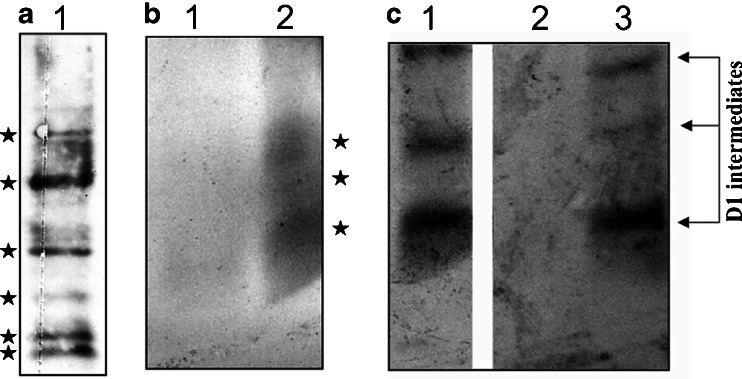
Immunochemical analysis of RNC complexes after double immunoprecipitation. **a** Western blot analysis of isolated RNC’s followed by the cross-linking with SPDP and then immunoprecipitation with antiserum directed against the cpSecY. For immunodetection, polyclonal antibody against cpSecY was used. **b** The proteins extracted from the high molecular mass complexes containing cpSecY and ribosomes were subjected to immunoprecipitation. Lanes 1 and 2 represent the precipitated products after two-step immunoprecipitation. The supernatant (lane 1) and pellet fraction (lane 2) were analyzed by immunoblotting with anti-cytochrome b_6_. Proteins subjected to PMF are indicated by *filled stars*. **c** Association of cpSecY and nasce chains with ribosomes. The proteins extracted from the high molecular mass complexes containing cpSecY and ribosomes, were subjected to immunoprecipitation with anti-D1, nascent D1 chains are indicated by an *arrow*. The precipitated products (lane 1) before second immunoprecipitation. Lanes 2 and 3 represent the precipitated products after two-step immunoprecipitation. The pellet (lane 2) and supernatant fraction (lane 3) were analyzed by immunoblotting with anti-D1. Proteins subjected to PMF are indicated by *filled stars*

Further evidence for the possible interaction between cytochrome b_6_ protein and cpSecY was obtained by subjecting the RNCs to a cleavable cross-linker (SPDP), followed by solubilization with SDS and immunoprecipitation with anti-cpSecY. Before the second immunoprecipitation, it was also verified by Western blotting that the cross-linked chloroplast RNCs contained the potentially interacting components, cpSecY, cytochrome b_6_ or D1 using antisera directed against these proteins (Fig. 7).

Precipitated cross-linked products were then cleaved with a reducing agent (DTT), solubilized with SDS or DM, and subjected to further precipitation under denaturing condition with anti-cytochrome b_6_ or anti-D1 cross-linked to protein A-Sepharose beads.

The precipitated products were centrifuged and separated by SDS-PAGE and subjected to Western blot (Fig. 7b, lane 1 and Fig. 7c, lanes 2 and 3).

Furthermore, the protein found in the stained band (marked by filled stars) was excised from a polyacrylamide gel and analyzed using MS and then the data were process using Mascot Distiller software. Output lists of precursor and product ions were compared to the National Center for Biotechnology database using the Mascot database search engine.

The D1 protein intermediates of different lengths were efficiently precipitated from supernatants (Fig. 7c, lanes 1 and 3). Indeed, the D1 intermediates can be cross-linked to cpSecY. The smeary band marked by filled stars could suggest the possible presence of cytochrome b_6_ proteins in immunoprecipitated complexes (Fig. 7b, lane 2). These bands were analyzed using mass spectroscopy with fingerprint analysis but no significant immunoprecipitation of nascent chains using cytochrome b_6_ antiserum was found, indicating that cpSecY did not interact with cytochrome b_6_ nascent chains released from the ribosome. The smeary band contained a mixture of various fragments that could not be clearly analyzed, and we did not observe any peptides indicated cytochrome b_6_.

The cytochrome b_6_ intermediate protein was not co-precipitated with anti-cpSecY followed by anti-cytochrome b_6_ (Fig. 7b lane 1 and 2), ruling out the interaction of cytochrome b_6_ with cpSecY.

## Discussion

The authors, have begun to experimentally address the mechanisms of targeting and insertion of chloroplast-encoded thylakoid membrane protein cytochrome b_6_ into the thylakoid membrane. These mechanisms still require further research. However based on a number of observations in chloroplasts, such as run-off translations of thylakoids with bound ribosomes and detection of translation intermediates in the membrane, it can be postulated that insertion of polytopic chloroplast-encoded membrane proteins occurs co-translationally (Klein et al. 1988; van Wijk et al. 1996).

Initiation of translation in the chloroplast occurs on 70S ribosomes, creating so-called RNC complexes. After initiation, elongation proceeds and the nascent chain emerges out of the ribosome tunnel (Nilsson et al. 1999). The observation of functional interaction of cpSecY with chloroplast 70S ribosomes suggested an involvement of cpSecY in the process (Zhang et al. 2001). The chloroplast cpSecY is common for the two targeting systems, the chloroplast-encoded SRP-dependent D1 protein insertion and SecA-mediated cytochrome f insertion. Two populations of cpSecY complexes are present in thylakoid membranes (Zhang et al. 2001). One population of approximately 100 kDa is likely to be involved in the posttranslational translocation of nuclear-encoded Sec-dependent substrates across the thylakoid membrane (Mori et al. 1999; Schuenemann et al. 1999). A second cpSecY population was associated with ribosomes (Zhang et al. 2001). Importantly, this portion of cpSecY was interacting with protein elongation intermediates and most probably forms the translocation channel where the chloroplast-encoded proteins integrate into the membrane.

Discontinuous translation of Dl may also be important for co-translational binding of cofactors to Dl and the insertion of this protein into the chloroplast thylakoid membrane (Kim et al. 1991).

This paper reports that several of the cytochrome b_6_ translation intermediates co-sediment with polysomes during sucrose cushion centrifugation. The 6–18 kDa cytochrome b_6_ translation intermediates were linked to isolated and immunoprecipitated biosynthetic ribosomes. The low level of mature cytochrome b_6_ is caused by poor co-sedimentation of cytochrome b_6_ protein with polysomes.

The authors argue that the 6–18 kDa polypeptides could be precursors of cytochrome b_6_, which were paused at discrete points during translation. The 6-kDa intermediates contain the first hydrophobic α-helices and other translation intermediates are also located near α-helices. This led the authors to examine if there was a relationship between the pause sites and synthesis of the four hydrophobic membrane-spanning α-helical domains of cytochrome b_6_. Ribosomes might pause at specific codons if cognate aminoacyl-tRNAs were limiting. However, analysis of the codons corresponding to pause sites revealed an inconsistent pattern. Similar observations were shown for D1 protein (Kim et al. 1991).

Ribosome pausing may also facilitate co-translational binding of co-factors such as chlorophyll to cytochrome b_6_ and aid the integration of cytochrome b_6_ into thylakoid membranes. Moreover, chlorophyll or β-carotene binding might alters ribosome pausing. Translational pausing in plastids concerns more than Dl protein. Translation intermediates have also been observed for the large subunit of ribulose-1,5 bisphosphate carboxylase (Mullet et al. 1986; Mühlbauer and Eichacker 1999). The feedback regulation of translation via ribosome tunnel interaction uses sequence information from the nascent chains and leads to transient translational pausing. Such a pausing can have beneficial effects on either the coordination of ribosome-associated factors or the co-translational folding of nascent peptides (Marin 2008).

As mentioned above, a cpSecY population was found to be associated with ribosomes. Importantly, this portion of cpSecY was interacting with D1 elongation intermediates and most probably forms the translocation channel where the chloroplast-encoded proteins integrate into the membrane (Zhang et al. 2001). A control experiment confirmed that D1 intermediates were stable and were co-immunoprecipitated with cpSecY. This corresponds to results obtained by Zhang et al. (Zhang et al. 2001), which was also in accordance with a previous study by Nilsson et al. (Nilsson et al. 1999).

The authors assumed that cpSecY could participate in cytochrome b_6_ integration, but results of this study show that the cytochrome b_6_ and its intermediates in RNCs may precipitate with the cytochrome b_6_ antibody but not co-precipitated with the cpSecY antibody.

The same result was obtained irrespective of the order of antibodies used. In the first case, antibodies against cytochrome b_6_ were used, and then antibodies against the cpSecY were applied (Fig. 6). In the second case, the above antibodies were used in the reverse order (Fig. 7).

These results indicate that cpSecY was not in the vicinity of cytochrome b_6_ intermediates during the elongation process and also did not act with the mature cytochrome b_6_ after translation.

A crucial process that occurs at the ribosome is the decision concerning the co-translational protein translocation into and across membranes versus folding in the cytosol. Co-translational protein translocation involves the interaction of SRP with ribosomes (Keenan et al. 2001), which allows SRP to recognize the hydrophobic signal sequence at the N termini of nascent chains as they emerge from the exit tunnel. The striking interaction between nascent chains and the ribosomal tunnel regulates the association of the SRP (Kramer et al. 2009).

Using cross-linking, the authors prove that cpSRP54 interacts specifically with the cytochrome b_6_ nascent chain only when it is attached to the 70S ribosome. This implies that cpSRP54 is involved in the biogenesis of cytochrome b_6_, however, only in the beginning of the elongation process.

The interaction was strongly dependent on the length of the nascent chain that emerged from the ribosome. Variations of the nascent chain showed that the affinity for cpSRP54 was lost when the nascent chain was ~120 amino acid residues long. cpSRP54 is lost during progressive elongation possibly via interaction with the other translocon proteins. cpSRP54 homologues in bacteria and ER of eukaryotes have been shown to interact predominantly with signal sequence or with hydrophobic transmembrane domains located at the N-terminus (Keenan et al. 2001; Nilsson and van Wijk 2002). cpSRP54 and cytochrome b_6_ interact during the early phase of cytochrome b_6_ elongation and this elongation requires hydrophobic domain. The hydrophobicity of the first 180 amino acids of cytochrome b_6_ is presented in Fig. SP2. These results suggest that cpSRP54 may not only assist in early steps of cytochrome b_6_ biogenesis but it can also be important in directing the first transmembrane domain of the cytochrome b_6_ to the SRP receptor, possibly Alb3. This type of interaction was observed for D1 protein (Nilsson and van Wijk 2002).

Some of the lower molecular weight products became more abundant when the RNCs were incubated prior to cross-linking. It is likely that they represent cross-links to as yet unidentified components and the cross-linked proteins are not specific. In these gel bands unknown proteins were identified by MS. Hence it would be interesting to investigate these proteins in future studies.

In conclusion, it is known that the translocation of chloroplast proteins into or across the thylakoid membrane occurs via a number of different pathways (Schuenemann 2007; Aldridge et al. 2009). There is still the question left if the chloroplast-encoded proteins also use several translocation pathways, or share only one common translocation apparatus.

The authors demonstrated that the cytochrome b_6_ nascent polypeptides complex is tightly associated with ribosomes. Additionally, the studies indicated that translation of cytochrome b_6_ was discontinuous. The causes of ribosome pausing and the functional significance of this phenomenon may be related to proper protein folding, insertion of cytochrome b_6_ into the thylakoid membranes, and the association of cofactors during this process as is the case for other proteins (Kim et al. 1991).

Structural and functional flexibility of the translocon is probably required for efficient protein folding and assembly during the biogenesis of multi-protein complexes, such as the cytochrome b_6_f complex or PSII and these processes are more complicated than it might first be expected. For instance, in *Arabidopsis*, LHCPs (light-harvesting chlorophyll proteins) is inserted into the membrane using an SRP/Alb3-dependent but cpSec-independent mechanism (Mori et al. 1999; Moore et al. 2003). However, it has also been shown that Alb3 is at least partially associated with the cpSecY translocase (Moore et al. 2003; Pasch et al. 2005), where it can be involved in the insertion or assembly of chloroplast-encoded thylakoid membrane proteins. Nevertheless, small quantities of LHCPs appeared to be inserted into the thylakoid membranes, even with the complete loss of Alb3. In the Alb3 mutant, LHCPs might be integrated into the thylakoid membrane spontaneously, or via the Sec-dependent pathway (Asakura et al. 2008).

However, cpSRP54 does not seem to be required for the targeting of all chloroplast-encoded thylakoid membrane proteins, since no interaction between cpSRP54 and the nascent chain of cytochrome f was observed. Instead, it has been shown that cytochrome f binds to cpSecA. Single ffc (∆cpSRP54) and chaos (∆cpSRP43) mutants demonstrated no defect in cytochrome f accumulation in the thylakoid membrane (Amin et al. 1999; Röhl and van Wijk 2001). Moreover, Asakura et al. suggest a functional link between cpFtsY and cpSecA/cpSecY translocation machinery for cytochrome f translocation (Asakura et al. 2008).

Further experiments are needed to analyze the targeting and insertion of the cytochrome b_6_ chloroplast-encoded thylakoid membrane protein, particularly the role of SRP and Alb3 proteins.

Co-translational protein targeting and insertion of membrane protein involves early interaction of RNCs with SRP. For conventional co-translational targeting of chloroplast-encoded substrate proteins, cpSRP54 engages with chloroplast ribosomes (Grudnik et al. 2009). Alb3 has been proposed to play a role in enabling Sec-dependent proteins to be co-translationally inserted into the thylakoid membrane (Wang and Dalbey 2011). The interaction of cpSRP with LHCP occurs via the cpSRP43 component. cpSRP enables Alb3 to insert LHCP into the thylakoid membrane. In mitochondria, Oxa1 (Alb3 homolog) facilitates the insertion of mitochondrial encoded proteins and certain nuclear-encoded proteins that are imported into the mitochondria and integrated into the inner membrane (He and Fox 1997). It acts as a translocase for the translocation of the N-tail of Cox II. In this pathway, Oxa1 interacts with Cox II in a co-translational manner and is believed to function not only as a translocase but also as a ribosome receptor (Wang and Dalbey 2011). Maybe similar pathways exist in chloroplasts and cytochrome b_6_ uses these for membrane integration in connection with cpSRP54.

These results should enable to identify more precisely the role of cpSRP54 and unravel the mechanisms of targeting, insertion, and assembly of thylakoid membrane proteins encoded by the chloroplast genome.

## Electronic supplementary material

Fig. SP1. Test of specificity of used antibodies. Fig. SP2. Hydrophobicity plots. The hydrophobicity of the first 180 amino acids of cytochrome b_6_ was calculated using the method of Engelman and Cornette. Table SP1. Identification of cytochrome b_6_ intermediates proteins by MS and PMF. Total coverage and peptide fragment matched in Mascot search for each protein intermediates.

ESM 1 (DOCX 822 kb)
