# Supplementary material for: Ribosome nascent chain complexes of the chloroplast-encoded cytochrome b6 thylakoid membrane protein interact with cpSRP54 but not with cpSecY
Source: J Bioenerg Biomembr. 2015 Jan 6;47(3):265–78. doi: 10.1007/s10863-014-9598-0 (PMC4555342; doi:10.1007/s10863-014-9598-0)
Supplement: Supplementary file 1 — (DOCX 822 kb) [file 10863_2014_9598_MOESM1_ESM.docx]

**Ribosome nascent chain complexes of the chloroplast-encoded cytochrome b_6_ thylakoid membrane protein interact with cpSRP54 but not with cpSecY.**

Małgorzata Piskozub^1^, Bożena Króliczewska^2^, Jarosław Króliczewski^1^.

^1^Faculty of Biotechnology, University of Wroclaw, Fryderyka Joliot-Curie 14a, 50-383 Wroclaw, Poland.

^2^Department of Animal Physiology and Biostructure, Wrocław University of Environmental and Life Sciences, Wrocław, Poland.

To whom correspondence should be addressed: e-mail: jakrol@windowslive.com

**Supporting information available**

Fig. SP1. Test of specificity of used antibodies. Fig. SP2. Hydrophobicity plots. The hydrophobicity of the first 180 amino acids of cytochrome b_6_ was calculated using the method of Engelman and Cornette. Table SP1. Identification of cytochrome b_6_ intermediates proteins by mass spectroscopy and peptide mass fingerprinting. Total coverage and peptide fragment matched in Mascot search for each protein intermediates.

**Fig. SP1.** Test of specificity of used antibodies. (A) Western blot analysis of total chloroplast protein used antibodies against: lane 1. NH2-terminus of cytochrome b_6_; lane 2. cpSecY; lane 3. D1 protein; (B) Western blot analysis of immunoprecipitated proteins from chloroplast by antibodies cross-linked to protein A Sepharose CL-4B. Lane 1. Molecular mass standards; lane 2. antibody against cpSecY; lane 3. antibody against D1 protein; lane 4. antibody against NH2-terminus of cytochrome b_6_; Detected bands was also analysed by mass spectroscopy.

A)

**
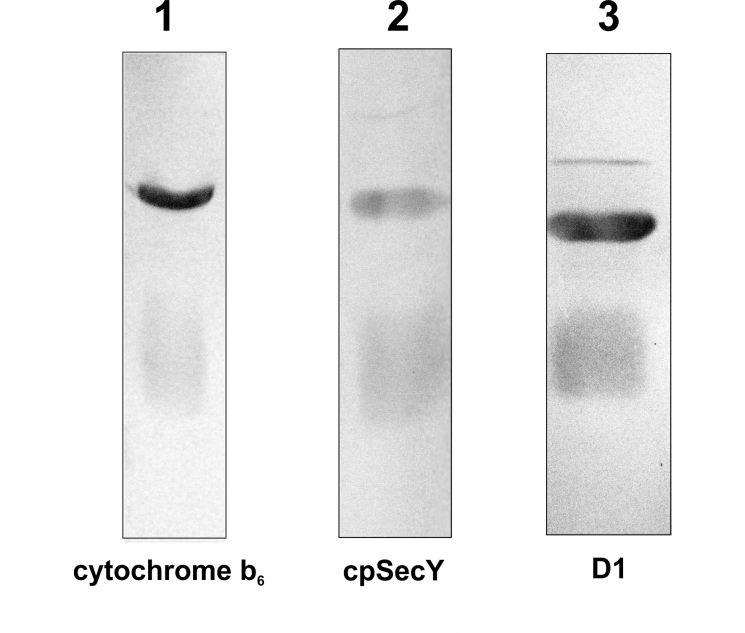
**

**B)**


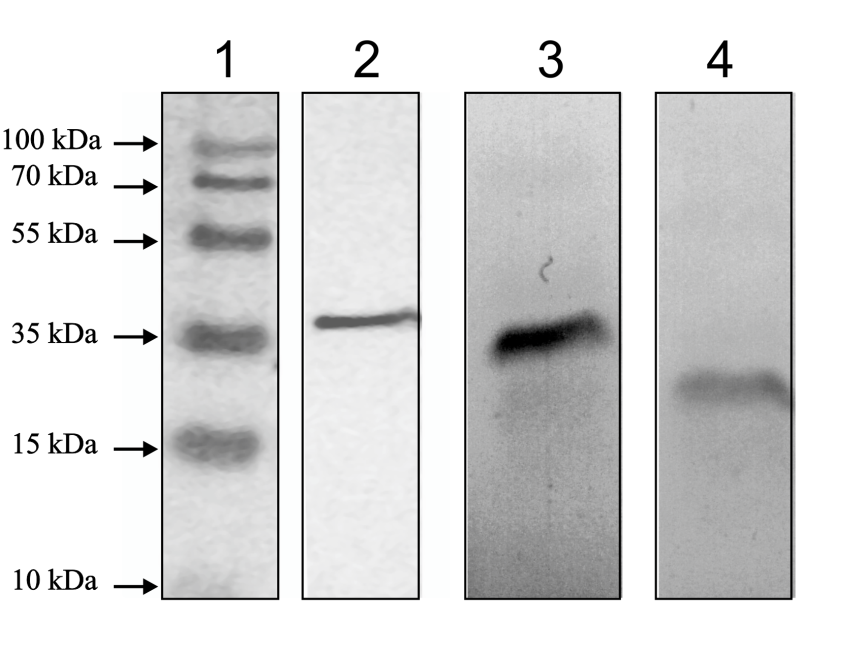


**Online Resource 2**

**Fig. SP2.** Hydrophobicity plots. The hydrophobicity of the first 180 amino acids of cytochrome b_6_ was calculated using the method of Engelman [[1](#_ENREF_1)] and Cornette [[2](#_ENREF_2)].

[1] Engelman, D.M., Steitz, T.A. and Goldman, A. (1986). Identifying nonpolar transbilayer helices in amino acid sequences of membrane proteins. Annu Rev Biophys Biophys Chem 15, 321-53.

[2] Cornette, J.L., Cease, K.B., Margalit, H., Spouge, J.L., Berzofsky, J.A. and DeLisi, C. (1987). Hydrophobicity scales and computational techniques for detecting amphipathic structures in proteins. J Mol Biol 195, 659-85.





**Table SP1.**

Identification of cytochrome b_6_ intermediates proteins by mass spectroscopy and peptide mass fingerprinting. Total coverage and peptide fragment matched in Mascot search for each protein intermediates.

| Protein (Da) | Total coverage % | Mass  (Da) | Peptide fragment matched* |
| --- | --- | --- | --- |
| **mcyt b_6_**  (24187) | 90 | 1143  6844  1952  884  3040  731 | VYDWFEER  YVPPHVNIFYCLGGITLTCF LVQVATGFAMTFYYRPTVTE AFASVQYIMTEANFGWLIR  WSASMMVLMMILHVFR  VYLTGGFK  FYSLHTFVLPLLTAVFMLMH FLMIR  QGIFGPL |
| **18 kDa**  (17851) | 88 | 1416  1952  3827  805 | LEIQAIADDITSK  WSASMMVLMMILHVFR  ELTWVTGVVLGVLTATFGVT GYSLPWDQIGYWAVK  FVTGVPDA |
| **14 kDa**  (13885) | 85 | 1143  1952  884  647 | VYDWFEER  WSASMMVLMMILHVFR  VYLTGGFK  ELTWV |
| **10 kDa**  (10428) | 93 | 1143  1416  6844  498 | VYDWFEER  LEIQAIADDITSK  YVPPHVNIFYCLGGITLTCF LVQVATGFAMTFYYRPTVTE AFASVQYIMTEANFGWLIR  SVHR |
| **6 kDa**  (6011) | 94 | 1143  1416  3257 | VYDWFEER  LEIQAIADDITSK  YVPPHVNIFYCLGGITLTCF LVQVATGFA |

*only chosen peptides with a mass bigger than ~500 Da and smaller than 7000 Da was shown. Coverage (%) was calculated for all well-identified peptides.
